# Metformin induces autophagy of cisplatin-resistant human gastric cancer cells in addition to apoptosis

**DOI:** 10.37796/2211-8039.1408

**Published:** 2023-06-01

**Authors:** Chih-Wun Fang, Jai-Sing Yang, Jo-Hua Chiang, Po-Chuen Shieh, Fuu-Jen Tsai, Chia-Wen Tsai, Wen-Shin Chang

**Affiliations:** a Division of Pharmacy, Zuoying Branch of Kaohsiung Armed Forces General Hospital, Kaohsiung, Taiwan; b Department of Medical Research, China Medical University Hospital, China Medical University, Taichung, Taiwan; c Department of Nursing, Chung-Jen Junior College of Nursing, Health Sciences and Management, Chiayi, Taiwan; d Department of Pharmacy, Tajen University, Pingtung, Taiwan; e School of Chinese Medicine, College of Chinese Medicine, China Medical University, Taichung, Taiwan; f China Medical University Children’s Hospital, China Medical University, Taichung, Taiwan

**Keywords:** Autophagy, Cisplatin-resistance, Gastric cancer, Metformin

## Abstract

Metformin has been used to treat cases of type 2 diabetes mellitus, and mounting studies have shown that metformin can act alone or in synergy with other anticancer agents to achieve anti-cancer efficacies on various types of tumors. However, the role of metformin in either inducing autophagy and cisplatin-resistance of human gastric cancer (GC) cells has never been examined. The study has established a cisplatin-resistant GC cell line and investigated the effects of metformin on inducing autophagy on it. The results demonstrated that treatment with metformin can concentration-dependently suppress the cell viability and cell confluence of cisplatin-resistant GC cells, while having no effects on human primary stomach epithelial cells (HPSEC). For the first time, we found that metformin can significantly increase the acidic vesicular organelles (AVO) level and decrease the acridine orange (AO) level spontaneously in the cisplatin-resistant GC cells. Thus, we further checked the other markers, Atg5, Atg12 and LC3-II, which showed that metformin indeed induced autophagy in the cisplatin-resistant GC cells. In addition, treatment of 3-Methyladenine (3-MA) can significantly rescue the metformin-induced autophagy. At the same time, metformin can induce the alterations of apoptosis-associated signal molecules, such as caspase-3 and caspase-7 activities. Overall, the pilot study provided evidence for metformin induced autophagy in addition to apoptosis, making it as an effective anticancer drug for the therapy of cisplatin-resistant GC. Killing the cisplatin-resistant GC cells with non-toxic metformin *via* both autophagy and apoptosis might extend its usefulness in our fighting with chemo-resistance of gastric cancer cells.

## 1. Introduction

Gastric cancer (GC) is still a critical cancer, with the 5th incidence and 4th mortality all over the world [1]. In Taiwan, GC is the 7th leading cause of cancer-associated mortality, and the mortality rate of GC was about 9.8 per 100,000 among the Taiwanese (https://www.mohw.gov.tw/cp-16-41794-1.html.). Most threatening information of GC is its high drug resistance rates and low survival rates, for instance, the overall 5-year survival rates of GC patients in the United States is lower than one thirds in 2018 (https://www.cancer.org/cancer/stomachcancer/detection-diagnosis-staging/survivalrates.html.). As for drug resistance, the situation is even worth, there is no any antidote or solution for cisplatin-resistant GC treatment, and it is an urgent quest for us to face the nightmare. In current clinical practice, there are several drugs suggested for GC patients, including carboplatin, cisplatin, paclitaxel, 5-fluorouracil, capecitabine and leucovorin [2]. However, none of them are satisfying since drug resistance often comes during the therapeutic period [3,4]. Therefore, translational scientists and clinical surgeons are keen to revealing a new strategy to conquer the problem of drug resistance.

Metformin is well accepted for the treatment of type 2 diabetes mellitus (DM) [2,5,6]. Molecularly speaking, inhibition of mitochondrial complex I and activation of AMP-activated protein kinase (AMPK) facilitates metformin to decrease the levels of glucose [7–9]. In addition, it is non-toxic for the normal cells and almost for the whole human bodies. From the viewpoint of anti-cancer, the decrease of ROS generation has been reported [7,8]. As for GC, some reported that metformin can restore microbiome diversity, which is closely related to *Helicobacter pylori* colonization and GC progression [10,11]. Metformin has been shown by at least three groups to suppress the cell proliferation in a panel of GC cell lines, including MKN45, MKN47, MKN-28, SGC-7901 and BGC-823, and cancer stem cells [12,13]. Metformin has also been shown to reduce the capacity of metastasis of human GC cells via inhibiting the progress of epithelial mesenchymal transition (EMT) [14,15]. However, up to now, not enough literature has revealed the effects of metformin on GC, and the anticancer mechanism has not been fully elucidated. It is only known that metformin seems to be capable of inhibiting the proliferative and metastatic behaviors of GC cells. We are interested in whether metformin could serve as a potential intervention for GC-drug resistance, and to reveal its potential in cisplatin-resistance GC treatment.

## 2. Materials and methods

### 2.1. Cell culture, chemicals and reagents

Metformin, thiazolyl blue tetrazolium bromide (MTT), In Situ Cell Death Detection kit, and all other chemicals and reagents mentioned in the study were purchased from Sigma–Aldrich (Merck KGaA, Darmstadt, Germany).

Trypsin–EDTA was obtained from Thermo Fisher Scientific (Waltham, MA, USA).

**Fig. 1:**
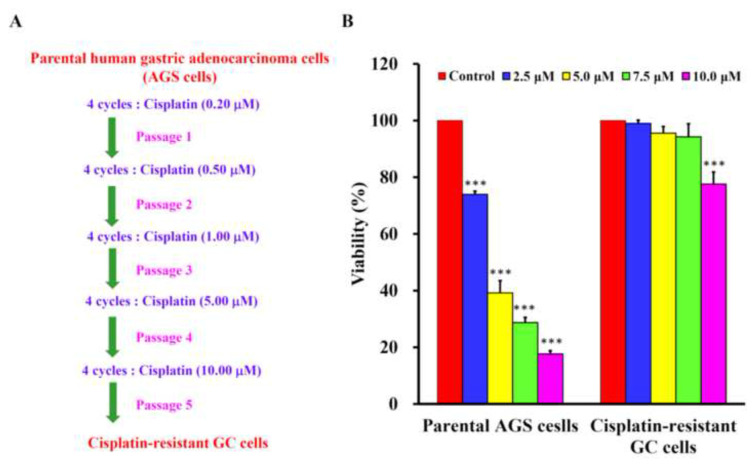
The establishment and identify of cisplatin-resistant gastric cancer cells. (A) Parental AGS cells were treated with consequentially increasing dose of cisplatin to establish the novel cell line. (B) Cell viability was individually measured by an MTT assay in parental AGS and cisplatin-resistant gastric cancer cells. The data were presented as the mean ± SD (n = 3). *** indicated significant different from the untreated control group with statistical P-value less than 0.001.

The novel cisplatin-resistant GC cells were developed by challenging the parental human AGS cell line (purchased from American Type Culture Collection, Manassas, VA, USA) with 0.2–10 μM of cisplatin treatments step by step as shown in Fig. 1. Typically, AGS and cisplatin-resistant GC cells were cultured in Dulbecco’s modified Eagle’s medium (DMEM) supplemented with 10% fetal bovine serum (FBS), 100 U/ml penicillin, 100 μg/ml streptomycin, 2 mM l-glutamine (Thermo Fisher Scientific, USA) and 10 μM of cisplatin in an incubator at 37 °C under humidified 5% CO_2_. Human primary stomach epithelial cells (HPSEC) purchased from Cell Biologics Company (Catalog No. H-6039; Chicago, IL, USA). HPSEC were cultured in T25 tissue culture flasks pre-coated with gelatin-based coating solution. HPSEC maintained and sub-cultured in Cell Biologics’ Culture Complete Growth Medium (Catalog No. H6621, Chicago, IL, USA) [2].

### 2.2. Cell viability and cell confluence assay

The cytotoxic effect of metformin was detected in an MTT assay, as previously published [16]. In concisely, the cells (1 × 10^4^ cells/well) were cultured in 96-well plates and exposed to indicate concentrations of metformin for 24 or 48 h. Following treatments, 10 μL MTT solution (5 mg/ml) was added per well, and cultured for an additional 3 h. Then the medium was totally removed, and the formation of formazan was solubilized using 100 μL dimethyl sulfoxide (DMSO). The absorbance was detected using an ELISA plate reader at 570 nm in a spectrophotometer.

For cell confluence assay, cisplatin-resistant GC cells (1 × 10^4^ cells/well) were seeded in 96-well plates and exposed to indicate concentrations of metformin for 0–48 h. Cells were visualized and photographed every 2 h with the IncuCyte S3 ZOOM (Essen Biosciences, Ann Arbor, MI, U.S.A.) microscope system [17].

### 2.3. Acridine orange (AO) staining

The acidic vesicular organelles (AVOs) formation of happens during cell autophagy. The cisplatin-resistant GC cells (2.5 × 10^5^ cells/well) were cultured in 24-well plates and exposed to Metformin (25 and 50 mM) for 48 h. Subsequently, cells were stained with 1 μg/ml Acridine orange (AO) for 30 min at 37 °C, Fluorescent microscope (Leica, Wetzlar, Germany) at 20 × magnification was used to visualize acidic vesicular organelles (AVOs), and analysis on the NucleoCounter NC-3000 using the built-in acridine orange (AO)/acidic vesicular organelles (AVOs) assay program as previously described [18,19].

### 2.4. Quantitative reverse transcription polymerase chain reaction analysis (RT-qPCR)

Quantitative reverse transcription polymerase chain reaction analysis (RT-qPCR) was performed as described previously [20]. The primer sequences for qPCR are as follows: human Atg5-F, 5′-tttcctccactgccatcattaa-3′; humanAtg5-R,5′-ggccaaaggtttcagcttca-3′; human Atg12-F, 5′-tgtggcctcagaacagttgttta-3′; human Atg12-R, 5′-cgcctgagacttgcagtaatgt-3′; human LC3-II-F, 5′-ccgaccgctgtaaggaggta-3′; human LC3-II-R, 5′-aggacgggcagctgctt-3′; human GAPDH-F, 5′-acacccactcctccaccttt-3′; human GAPDH-R, 5′-tagccaaattcgttgtcatacc-3′. Relative mRNA quantification was normalized to the mRNA expression levels of GAPDH [21,22].

### 2.5. Terminal deoxynucleotidyl transferase-mediated dUTP nick end labeling (TUNEL) analysis

The cisplatin-resistant GC cells (2.5 × 10^5^ cells/well) were cultured in 6-well plates and exposed to Metformin (25 and 50 mM) for 48 h. The cells were stained with the *In Situ* Cell Death Detection Kit, Fluorescein (Sigma–Aldrich; Merck KGaA), according to the manufacturers’ instruction as we previously published [18].

### 2.6. Caspase-3/-7 activity assay

The cisplatin-resistant GC cells (2.5 × 10^5^ cells/well) were cultured in 6-well plates and exposed to Metformin (25 and 50 mM) for 48 h. The cells were harvested by centrifugation at 400×*g* prior to incubation with the working solution provided and analysis on the NucleoCounter NC-3000 using the *Caspase-3/-7* assay program as previously described [18,23].

### 2.7. Statistical analysis

All results are presented as the mean ± standard deviation for each indicated treatment with multiple repeats. The data were statistically analyzed by one-way analysis of variance followed by Dunnett’s test using SPSS software version 16.0 (SPSS, Inc., Chicago, IL, USA). All the counterpart data were subject to unpaired *Student’s t*-test for comparison between the two groups, while any *P*-value <0.05, <0.01, and 0.001 were considered to be statistically significant and marked with *, ** and *** in the figures, respectively [20,24].

## 3. Results

### 3.1. Cisplatin was less cytotoxic to cisplatin-resistant GC cells than the parental AGS cells

The cisplatin-resistant GC cells were established successfully (Fig. 1A). After the parental AGS cells and cisplatin-resistant GC cells were treated with 2.5, 5, 7.5 and 10 μM cisplatin for 24 h, the MTT assay was adapted to analyze the viability of the parental AGS cells and cisplatin-resistant GC cells. The results showed that cisplatin can significantly reduce the cell viability of parental AGS cells after incubation concentration-dependently at 2.5, 5, 7.5 and 10 μM (Fig. 1B). The IC_50_ of cisplatin for parental AGS cells and cisplatin-resistant GC cells were 3.75 ± 1.24 μM and 24.69 ± 3.50 μM, respectively.

### 3.2. Metformin was cytotoxic to cisplatin-resistant GC cells, but neither parental AGS cells nor primary stomach epithelial cells

**Fig. 2:**
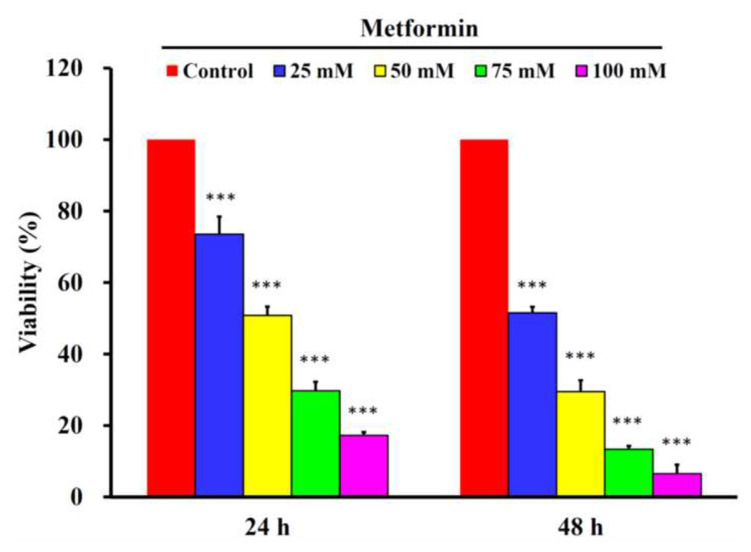
The effects of metformin on cell viability of cisplatin-resistant gastric cancer cells. The cisplatin-resistant gastric cancer cells were treated with 0, 25, 50, 75 and 100 mM of metformin for 24 and 48 h, and then subject to MTT for analyzing their cell viability. The data were presented as the mean ± SD (n = 3). *** indicated significant different from the untreated control group with statistical P-value less than 0.001.

**Fig. 3:**
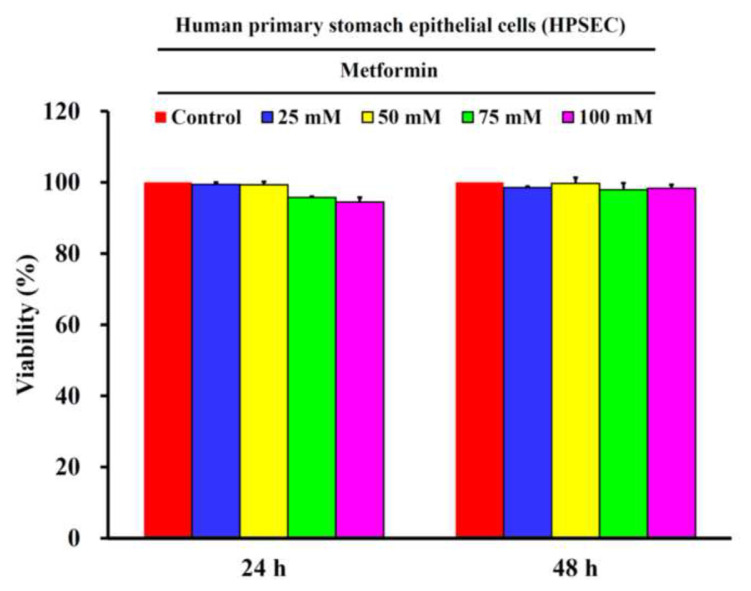
The effects of metformin on cell viability of human primary stomach epithelial cells (HPSEC). The HPSEC cells were treated with 0, 25, 50, 75 and 100 mM of metformin for 24 and 48 h, and then subject to MTT for analyzing their cell viability. The data were presented as the mean ± SD (n = 3). *** indicated significant different from the untreated control group with statistical P-value less than 0.001.

Metformin suppressed the cell viability of both parental AGS cells [2] and cisplatin-resistant GC cells (Fig. 2). In detail, metformin suppressed the cell viability of cisplatin-resistant GC cells concentration-dependently from 25 mM to 100 mM at 24 and 48 h, respectively. The treatments of metformin suppressed 73.52 ± 4.93%, 50.81 ± 2.50%, 29.71 ± 2.52% and 17.26 ± 0.92% of cell viability of cisplatin-resistant GC cells at 24 h, while 51.55 ± 1.68%, 29.47 ± 3.20%, 13.36 ± 0.95% and 6.51 ± 2.53% of cell viability of cisplatin-resistant GC cells at 48 h (Fig. 2). Generally speaking, metformin significantly suppressed the cell viability of cisplatin-resistant GC cells concentration- and time-dependently. As for the primary cultured stomach epithelial cells (HPSEC), there was no significant cytotoxicity found at any dosage of metformin tested from 25 mM to 100 mM at 24 and 48 h (Fig. 3).

### 3.3. Metformin suppressed cell confluence on cisplatin-resistant GC cells

**Fig. 4:**
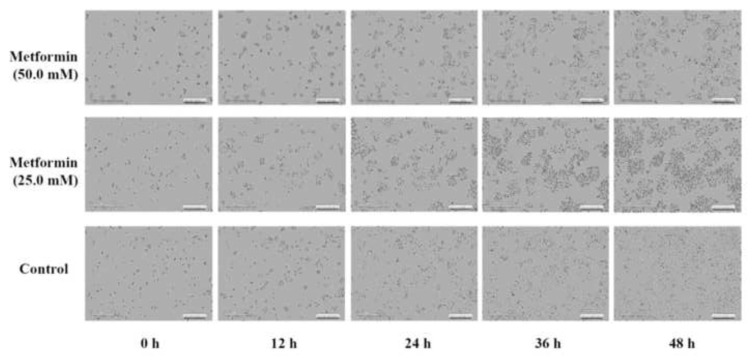
The effects of metformin on cell confluence of cisplatin-resistant gastric cancer cells. The cisplatin-resistant gastric cancer cells were treated with 25 and 50 mM of metformin for 0, 12, 24, 36 and 48 h, and then subject to IncuCyte S3 ZOOM microscope observation for their cell confluence.

In Fig. 4, under the IncuCyte S3 ZOOM microscope observation, the continuous confluence of cisplatin-resistant GC cells treated with 0, 25, and 50 mM of metformin were shown (Fig. 4). The bottom panel showed that the cisplatin-resistant GC cells grew at 0, 12, 24, 36 and 48 h. The middle and top panels showed that the cisplatin-resistant GC cells treated with 25 and 50 mM of metformin and grew at 0, 12, 24, 36 and 48 h, respectively. The number of cells at each time point was less than the counterpart at the bottom concentration-dependently (Fig. 4).

### 3.4. Metformin can induce cisplatin-resistant GC cells to undergo autophagy

**Fig. 5:**
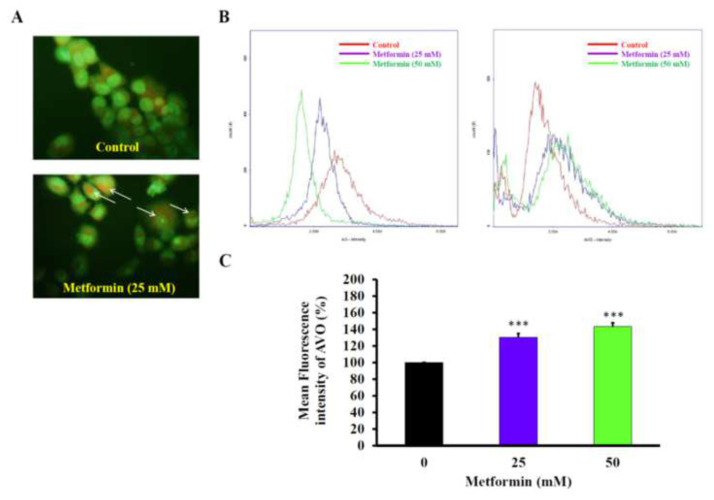
The effects of metformin on autophagy of cisplatin-resistant gastric cancer cells. The cisplatin-resistant gastric cancer cells were treated with 25 (A) and 50 (A and B) mM of metformin for 48 h, and then subject to (A) microscope observation for the appearance of autophagy. (B and C) detection of alterations of acidic vesicular organelles (AVO) and acridine orange (AO) levels in the cisplatin-resistant gastric cancer cells.

**Fig. 6:**
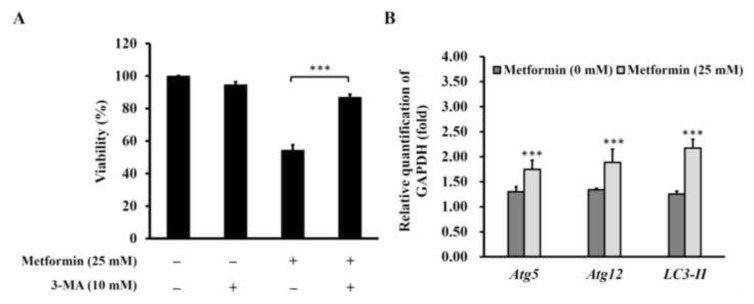
The confirmation of metformin on inducing autophagy in cisplatin-resistant gastric cancer cells. (A) The cisplatin-resistant gastric cancer cells were treated with metformin (25 mM), 3-MA (10 mM) or metformin plus 3-MA for 48 h, and then subject to MTT for analyzing their cell viability. The data were presented as the mean ± SD (n = 3). *** indicated significant different from the untreated control group with statistical P-value less than 0.001. (B) The cisplatin-resistant gastric cancer cells were treated with or without metformin (25 mM), and then the autophagy markers, atg5, atg12, and LC3-II were measured by qPCR for thrice. GAPDH serve as an internal standard for quantitation.

We used acridine orange (AO), which accumulated in a protonated form inside acidic vesicular organelles (AVO) to investigate whether metformin induced autophagy in the established cisplatin-resistant GC cells. Under the treatments of 25 and 50 mM of metformin, AO decreased (Fig. 5A and B) while AVO increased (Fig. 5C) concentration-dependently in the cisplatin-resistant GC cells. The novel finding of metformin induced autophagy in the cisplatin-resistant GC cells was confirmed by treating the metformin-induced autophagy cisplatin-resistant GC cells with or without 3-methyladenine (3-MA), an autophagy inhibitor for 48 h. The results showed that 3-MA could rescue the metformin-induced loss of cell viability in cisplatin-resistant GC cells (Fig. 6A). In addition, treatment of 25 mM of metformin could induce several autophagy markers, including Atg5, Atg12, and LC3-II mRNA (Fig. 6B).

### 3.5. Metformin can promote cisplatin-resistant GC cells to undergo apoptosis

**Fig. 7:**
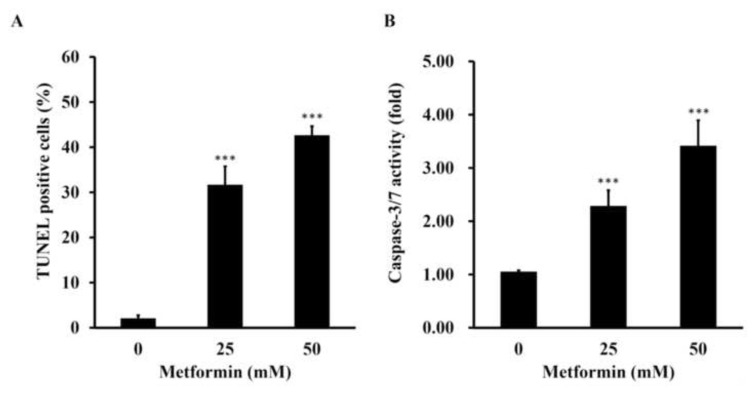
The confirmation of metformin on inducing apoptosis in cisplatin-resistant gastric cancer cells. (A) The cisplatin-resistant gastric cancer cells were treated with 0, 25 and 50 mM of metformin for 48 h, and then subject for TUNEL assay to investigation the appearance of apoptotic DNA strand breaks. (B) The cisplatin-resistant gastric cancer cells were treated with 0, 25 and 50 mM of metformin for 48 h, and then subject for caspase-3/-7 activity assay to investigate the alterations of apoptotic related caspase activating signals.

Then, 25 and 50 mM of metformin were examined of its capacity to induce cisplatin-resistant GC cells to undergo apoptosis with TUNEL assay (Fig. 7A) and caspase-3 and caspase-7 activities (Fig. 7B). The results showed that metformin was capable of inducing the cisplatin-resistant GC cells to undergo apoptotic cell death.

### 3.6. Metformin can activate the activation of phosphorylated AMPK in cisplatin-resistant gastric cancer cells

**Fig. 8:**
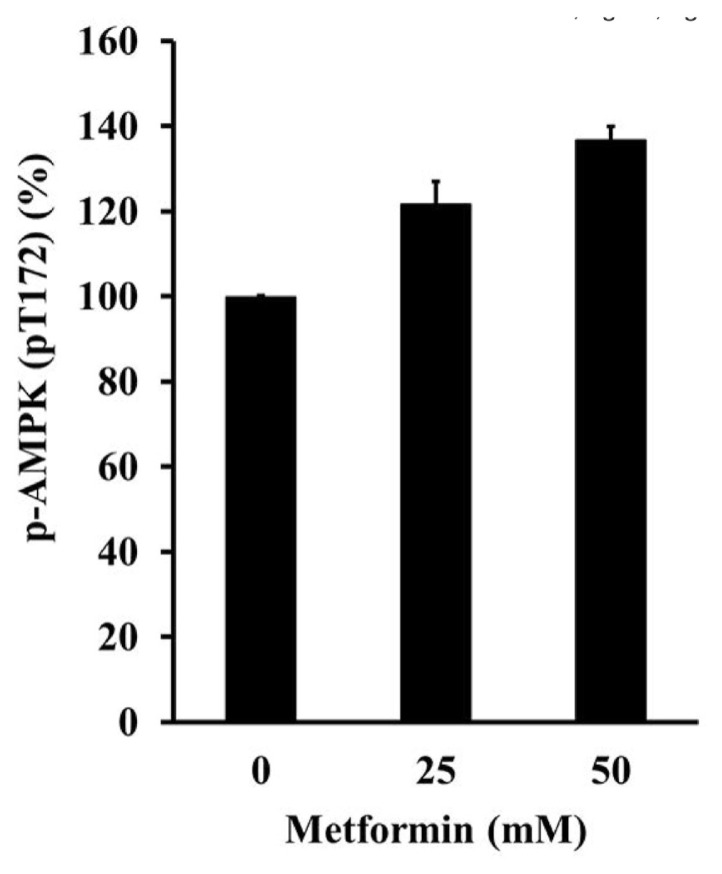
The activation of phosphorylated AMPK in cisplatin-resistant gastric cancer cells. The cisplatin-resistant gastric cancer cells were treated with 0, 25 and 50 mM of metformin for 48 h, and then subjected to investigate the expression of phosphorylated AMPK.

Last, we are interested in the activation of metformin’s activity to enhance the expression level of phosphorylated AMPK in cisplatin-resistant GC cells (Fig. 8). The results showed that metformin was capable to activate phosphorylated AMPK in cisplatin-resistant GC cells dose-dependently at 25 and 50 mM.

## 4. Discussion

Metformin, a commonly used T2DM drug, has been shown to inhibit the proliferation and induce apoptosis in various types of cancer [2,5,11]. However, it is not yet known that whether metformin is capable of inducing autophagy in GC cells, especially those drug resistant ones. Thus, we have established a new human cisplatin-resistant GC cells from an existing AGS cancer cell line (Fig. 1A), and examine whether metformin can induce autophagy in this cell line or not. The pilot data have checked its cisplatin resistance (Fig. 1B), confirmed that metformin induced apoptosis (Fig. 7), and found that metformin induced autophagy (Figs. 5 and 6).

In the year of 2020, Liu and his colleagues reported that metformin can suppress the progress of GC via multiple endpoints, including inhibiting the cell proliferation, migration and invasive capacity [25]. The key point is that metformin can induce autophagy in a panel of GC cell lines, MKN45, HGC27, SGC7901, BGC823, N87, SNU216 and MGC803 cells [25]. It is very exciting that the efficacy of metformin to induce autophagy seems to fit various types of GC cells. In this study, we have extended its capacity of induce autophagy in cisplatin-resistant cells. In the near future, it is worthy of revealing whether metformin can inhibit the GC progress in resistant cell lines for other chemotherapy agents used in clinical GC therapy, such as carboplatin paclitaxel, 5-fluorouracil, capecitabine and leucovorin.

Similar to GC, diabetes mellitus (DM) is an increasingly prevalent global disease that closely related to many other diseases [26]. For instance, mounting studies have demonstrated that DM predisposes its cases to a higher risk of cardiovascular diseases and various types of cancer [27,28]. Relationship between GC and DM remains unclear since contradictory results have been produced from various studies [27,29–32]. In 2017, some reported that DM incidence was associated with higher mortality rates for GC with a meta-analysis study [6]. In 2019, Cheung and his colleagues reported that DM may promote the incidence of GC by 67% [11]. Several mechanisms through which DM promotes GC development such as hyperglycemia-induced DNA damage, increased production of reactive oxygen species (ROS), and stimulation of cell proliferation and angiogenesis due to altered glucose metabolism have been reported [6,11,31]. However, the differences among various populations, cultures, GC etiology, and genetic backgrounds still in need to examine.

The intracellular mechanisms of metformin are mostly elucidated, at least from viewpoint of anti-DM [33–36]. Since the most prevalent regions of GC located in Eastern countries, the influence of metformin on GC with the overall analysis of T2DM patient population in the National Health Insurance Database of China, Korean, and Taiwan are beneficial for the Eastern GC patients. From a published pilot study, it is reported that metformin therapy for more than three years may contribute to a significant lowering of GC risk by 43%, comparing those with metformin therapy [37–39]. Although the detail mechanism of why and how metformin do good to prevent elevated GC incidence, the epidemiological study has shown that metformin works. In addition, mounting studies supported the idea that metformin is associated with a decreased incidence and mortality of cancer among T2DM patients [40].

Not only GC, metformin has also been showed to inhibit the progress of lung cancer, breast cancer, colorectal cancer, liver cancer, pancreatic cancer, cervical cancer, endometrial cancer, ovarian cancer, prostate cancer, and renal cancer [41–50]. Most evidence demonstrated that the anticancer mechanisms of metformin are mainly mediated via the AMPK/mammalian target of rapamycin (mTOR) signaling [51,52]. We have also examined the mechanisms in charge of metformin induced autophagy, the results showed that phosphorylated AMPK (pT172) was elevated by 25 and 50 mM metformin treatment (Fig. 8). The exact role of phosphorylated AMPK was not identified for its involvement of apoptosis or autophagy or both. Further signaling network investigations can be conducted in the near future.

## 5. Conclusion

In summary, our findings suggest that treatment of cisplatin-resistant GC cells with metformin can induced autophagy in addition to apoptosis at a clinical available dosage. Interestingly, the dosage is less-toxic to human normal gastric epithelial cells. Further investigations are needed to complete the signaling network about the autophagy we proposed in the present pilot study. Our studies provide the extended insight for metformin’s potential therapeutic application in treating those cisplatin-resistant GC patients.
